# Antioxidant efficiency of *Sarcophyton* crude extract against gentamicin toxicity in male albino rats

**DOI:** 10.1038/s41598-025-90316-5

**Published:** 2025-03-03

**Authors:** Aml Talaat, Nada S. Badr, Aml Z. Ghoneim, Salwa A. El-Saidy

**Affiliations:** Zoology Department, Faculty of Science, Damanhour University, Damanhour, Egypt

**Keywords:** Gentamicin, *Sarcophyton*, Spleen and liver, Oxidative stress, Total antioxidant capacity, DNA fragmentation, Physiology, Zoology

## Abstract

Gentamicin is an antibiotic widely used in treating bacterial infections. However, it’s clinical interest is limited by it’s toxic side effects on vital organs. *Sarcophyton* soft coral is a source of natural products with a range of bioactivities. As such, the objective of this work was to assess how well *Sarcophyton* extract mitigated the gentamicin toxicity in rats. Four equal groups, each with five adult male albino rats, were randomly assigned: the control group, the *Sarcophyton* group given a *Sarcophyton* extract dose of 200 mg/kg/day orally for seven days, the gentamicin group receiving an intraperitoneal gentamicin dose of 100 mg/kg/day for seven days, and the combined administration group. Rats that received gentamicin injections saw a fall in body weight along with a decrease in liver function and all hematological parameters except the white blood cell count. The tissue’s total antioxidant capacity (TAC) dropped as a result of gentamicin, indicating oxidative stress. Gentamicin additionally caused histological alterations and significant increases in DNA fragmentation levels in the spleen and liver tissues. In contrast, the combined administration of gentamicin and *Sarcophyton* extract preserved body weight, maintained liver tissue structure and function, and improved hematological markers. Moreover, it strengthened the tissue’s TAC, restored the normal structure of the spleen tissues, and decreased the tissue’s DNA fragmentation. *Sarcophyton*’s chemical components, identified using gas chromatography-mass spectrometry, have hepatoprotective, antioxidant, and anti-inflammatory qualities, which are responsible for the extract’s ameliorative effects. Finally, *Sarcophyton* extract is a natural medication that may help reduce the toxicity caused by gentamicin.

## Introduction

Aminoglycoside antibiotics, mainly gentamicin, are used in the treatment of bacterial infections in humans and animals, especially those caused by aerobic gram-negative bacteria^1^. Nevertheless, gentamicin’s severe adverse effects on the body’s major organs, such as the kidney, spleen, and liver, through the generation of oxidative stress, the depletion of antioxidant defenses, and the stimulation of inflammation, limit it’s therapeutic utility^2,3^. The spleen is considered the largest secondary immune organ in the body and is responsible for blood filtration^4^. The liver is the most responsible organ for the detoxification and elimination of xenobiotics from the body^5^. Because of this, the spleen and liver are exposed to a variety of harmful substances that can cause toxicity^6,7^. Hepatotoxicity and hematotoxicity are considered to be among the most dangerous gentamicin toxic effects brought on by inflammation and production of reactive oxygen species (ROS), which are able to induce apoptosis in liver cells and red blood cells, resulting in hepatic failure and anemia^8^.

The generation of ROS by gentamicin leads to necrosis and cellular damage through a number of pathways, including lipid peroxidation, protein oxidation, and DNA fragmentation^1^, both of which impair the activity of several antioxidant enzymes, including catalase and superoxide dismutase, and cause mitochondrial dysfunction^8^. Additionally, gentamicin amplifies the activation of nitrosative tissue stress and inflammatory markers and modulates the expression of the caspase family and mitogen-activated protein kinases^9^. Thus, significant attention has been paid to natural sources to ameliorate the side effects caused by gentamicin.

*Sarcophyton *(order: Alcyonacea, family: Alcyoniidae) is one of the most common soft corals in the Red Sea^10,11^. It is acknowledged as a plentiful natural source of bioactive substances, including steroids and terpenoids^12–14^. These bioactive compounds represent the main chemical defense mechanism for soft corals to protect them from natural predators^15^. The wide spectrum of therapeutic properties of *Sarcophyton *steroids and terpenoids encourages an inquiry into their ability to mitigate the harmful effects caused by gentamicin, as these compounds are well-known as anti-inflammatory agents^14,16,17^. Moreover, *Sarcophyton *steroids exhibit a potent antioxidant action by scavenging active free radicals, inhibiting lipid peroxide formation, and regulating the activities of glutathione S-transferases and superoxide dismutase enzymes, and consequently protect the cells from injury^17^. *Sarcophyton *extracts were used in a wide range of interesting pharmacological bioactivities, including hepatoprotective^11,17,18^, anti-proliferative^19^, antioxidant^20^, antimicrobial^21,22^, and anti-inflammatory activities^23^. Since gentamicin is still used for it’s effective activity in eliminating bacteria despite it’s harmful effects, it was necessary to search for a treatment that would limit these harmful effects and take full advantage of gentamicin without causing toxicity to other organs. As far as we are aware, no research has been done on the effects of Red Sea *Sarcophyton* soft coral extract on the spleen and liver against gentamicin toxicity. Thus, this investigation is suggested for screening the bioactive chemicals of the Egyptian soft coral *Sarcophyton* and ascertaining whether it has a mitigating effect on the gentamicin-induced toxicity in the rats’ spleen and liver.

## Materials and methods

### Chemicals and drugs

Gentamicin (Garamycin): Ampoules (80 mg/2 ml) obtained from the Egyptian Drug Store. The remaining chemicals were of analytical quality and were acquired from reputable commercial vendors.

### ***Sarcophyton ***collection and identification

**Fig. 1:**
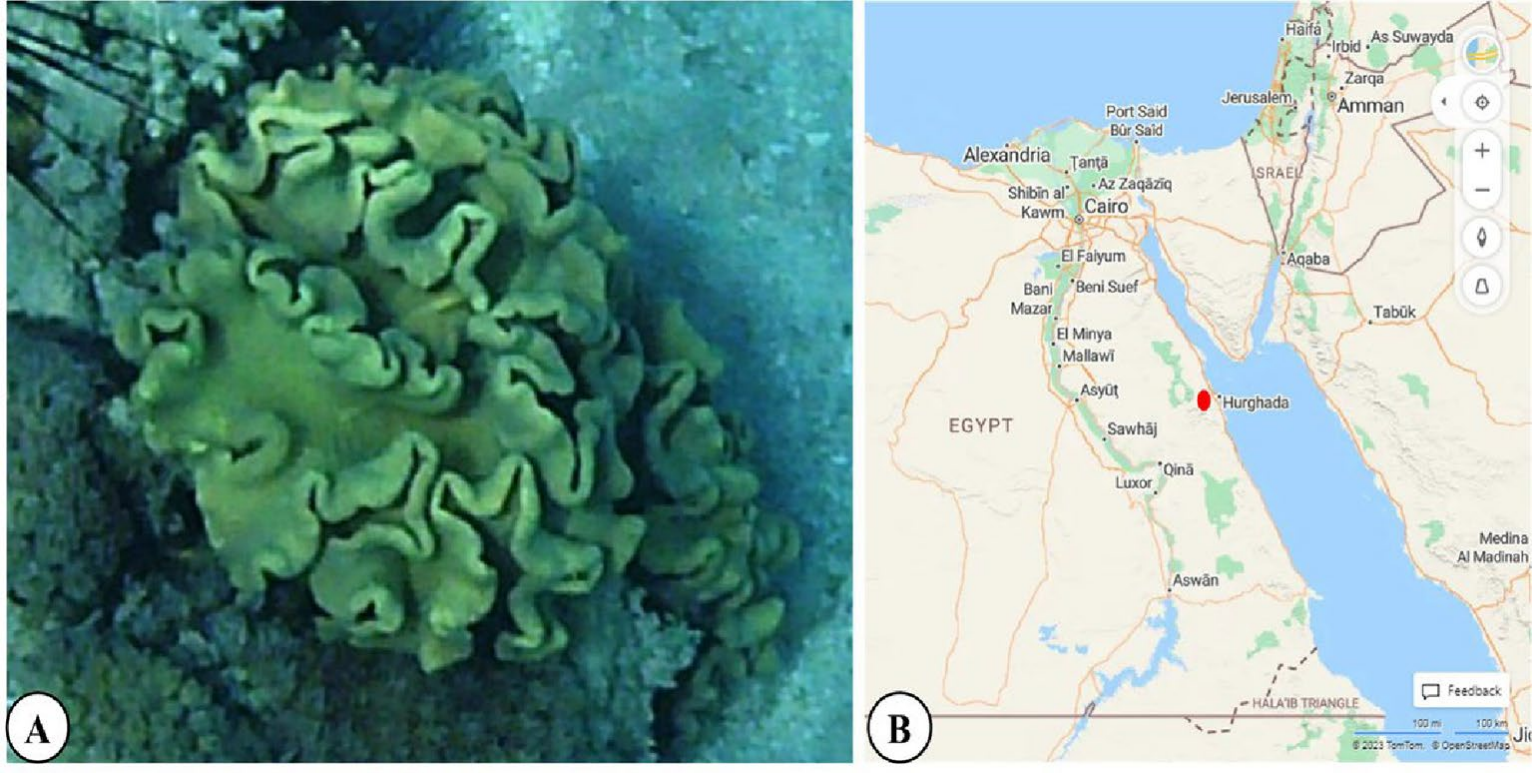
** (A)** *Sarcophyton* soft coral and **(B)** A map showing the sampling site, Hurghada (red circle), on the Egyptian Red Sea coast, from which soft coral was collected (https://mapmaker.tomtom.com/, version = 2.1).

The soft coral *Sarcophyton* was collected from Hurghada on the Egyptian Red Sea coast **(Fig. 1)**. Sampling was performed using the self-contained underwater breathing apparatus (SCUBA) diving technique at a depth of 3–6 m. *Sarcophyton* was identified according to **Fabricius and Alderslade**^24^ and **Janes and Leewis**^25^ based on the shape of the interior sclerites. The samples were washed with distilled water and frozen for further analysis.

### Preparation of the *Sarcophyton* crude extract

The small pieces of the air-dried sample were extracted by maceration in absolute methanol (1:2, *w/v*) till exhaustion, with occasional stirring. After passing through the Whatman No. 1 filter paper, the methanolic extract was concentrated at 30 to 40 °C in a vacuum rotary evaporator and then lyophilized to obtain a dry residue^11,26^. The crude extract, with a yield percentage of 6.43% of the original weight of the sample, was stored at ^_^80 °C until subsequent processing.

### Identification of the ***Sarcophyton*** crude extract’s bioactive compounds utilizing gas chromatography-mass spectrometry (GC-MS)

In line with a technique outlined by **Ismail et al.**^27^. with slight modifications, gas chromatography-mass spectrometry (GC-MS) analysis was conducted on *Sarcophyton* crude extract using a GC-MS spectrometer (Perkin Elmer model: Clarus 580/560S) equipped with an Elite-5MS column (30 m length, 250 μm internal diameter, and 0.25 μm df). The instrument was initially set to a temperature of 80 °C for 8 min before increasing to 260 °C. The GC-MS analysis was performed by injection of 1 µl of sample at an injection temperature of 280 °C, with a solvent delay time of 6 min, a transfer temperature of 150 °C, a source temperature of 200 °C, and a split ratio of 20:1. The mass spectrometer was set to operate in electron impact mode at 70 eV and to scan a range of values from 40 to 550 Da. High-purity helium was used as the carrier gas and was pressurized to 2223 psi, and the gas flow rate was set to 122 ml/min. The chemical constituents of the extract were identified by comparing the resulting mass spectra with those in a mass spectral library.

## Experimental animals

Twenty pathogen-free adult male albino Wistar rats (*Rattus norvegicus albinus*) in good health were utilized in this investigation. They were acquired from the Animal House Colony of the National Research Centre in Dokki, Giza, Egypt. The rats possessed an average weight of 128.83 ± 4.60 g at the beginning of the experiment and an age of approximately 2–3 months. The experiment began at least two weeks after the animals were housed in an animal holding room for acclimatization. The rats were kept in natural settings at 25 ± 2 °C, 60–70% relative humidity, and plenty of natural light. The rats were kept in hygienic, spacious plastic cages that were cleaned every day, with five rats in each cage. The animals had unlimited access to food and water throughout the experiment.

## Ethics approval

The experiment was conducted in accordance with ARRIVE guidelines for the care and use of laboratory animals, which were authorized by the Faculty of Science at Damanhour University in Egypt (Ethical Approval No. DMU-SCI-CSRE-231103). Every attempt was made to minimize the amount of suffering endured by the animals and to use fewer of them.

## Experimental design

Four experimental groups were randomly selected from the twenty male albino rats used in this investigation (5 rats per group). The groups were assigned as follows:

1. Control group (GI): Rats in this group received no treatment.
2. *Sarcophyton* group (GII): This group of rats orally received *Sarcophyton *methanolic extract at a dose of 200 mg/kg/day for seven days^11^. The desired dose of *Sarcophyton* extract was prepared with distilled water.
3. Gentamicin group (GIII): The rats in this group were given an intraperitoneal injection of gentamicin at a hazardous dose of 100 mg/kg/day for seven days^28^.
4. Combined administration group (GIV): This group of rats was given gentamicin (100 mg/kg) intraperitoneally and *Sarcophyton* methanolic extract (200 mg/kg) orally for seven days.

## Determination of body weight changes

The rats were fasted overnight on the 7th day of the experiment, and the final body weights (g) of the rats were recorded on the 8th day of the experiment before euthanizing. The following formula was used to get the percentage of body weight change:

Body weight change percentage (%) = (final weight – initial weight)/initial weight × 100.

### Sample collection

On the eighth day, twenty-four hours after their last dosage, sodium pentobarbital (40 mg/kg) was infused intraperitoneally to anesthetize the animals in each group. Through the use of a capillary tube, blood samples were taken from the retroorbital plexus and placed into EDTA tubes (KemikoVacutainer, Egypt) for hematological measurements and plain tubes to measure biochemical parameters. The samples were allowed to clot and centrifuged for ten minutes at three thousand rpm to extract clear serum. After being kept at − 20 °C, the serum was utilized to measure biochemical parameters. After blood sample collection, rats were euthanized by cervical dislocation for tissue sample collection. The spleen and liver of the rats in each experimental group were carefully excised and washed with saline. For additional biochemical studies, portions of the spleen and liver were frozen at − 20 °C, while the remaining tissues were preserved in 10% neutral formalin for histological investigations.

## Hematological parameters

Automated hematology analyzers (8000i, Sysmex, Japan) were used to measure complete blood counts (CBCs), which include the count of red blood cells (RBCs), hemoglobin (Hb), hematocrit (HCT), mean corpuscular volume (MCV), mean corpuscular hemoglobin (MCH), mean corpuscular hemoglobin concentration (MCHC), the count of platelets (Plts), and the count of white blood cells (WBCs).

## Biochemical parameters

### Liver enzyme activity

The colorimetric method of **Reitman and Frankel**^29^ was utilized to determine the activities of aspartate amino transaminase (AST) and alanine amino transaminase (ALT) using a colorimetric kit supplied by the Egyptian Bio-diagnostic Company (CAT. No. AS 10 61 (45) for AST and CAT. No. AL 10 31 (45) for ALT).

### Lipid peroxidation

After homogenizing portions of the spleen and liver tissues in 10% *w/v* ice-cold sodium-potassium phosphate buffer (0.01 M, pH 7.4), the mixtures were centrifuged for fifteen minutes at 4 °C and 9000 rpm. As per the manufacturer’s protocol of the Egyptian Bio-diagnostic Company (CAT. No. MD 25–29), the obtained supernatant was used for lipid peroxidation evaluation. This was measured by determining the concentration of malondialdehyde (MDA) using thiobarbituric acid reactive substances (TBARS) in accordance with the colorimetric method described by **Ohkawa et al.**^30^.

### Determination of total antioxidant capacity (TAC)

Using the Egyptian Bio-diagnostic Company’s colorimetric kit (CAT. No. TA 25 13), the TAC of the spleen and liver tissues was determined using **Koracevic et al.**'s technique^31^.

### Histological study

Sections of the spleen and liver were preserved in 10% neutral formalin, subsequently dehydrated using increasing concentrations of ethyl alcohol, and finally cleaned in xylol. Specimens were embedded in melted paraffin wax with a melting point of 58 °C. After cutting sections to 4 μm thick using a microtome (Leitz 1512, Leitz, Wetzlar, Germany), the sections were placed on sanitized glass slides and stained with hematoxylin and eosin (H&E)^32^. A light microscope (Olympus, Tokyo, Japan) with 10X and 40X objective lenses was used to view and take pictures of the stained slides.

### Morphometric study of the spleen

Measurements of the capsule thickness (µm) along with lymphatic follicle diameter (µm) and surface area (µm^2^) were made using H&E-stained spleen sections at 400X magnification. The Image J program (RRID: SCR_003070) was used to carry out these measurements^33^.

### Histopathological scoring analysis of liver sections

The histopathological score was evaluated using the methodology proposed by **Ghoneum and El-Gerbed**^34^. For each animal group, at least three slides, with ten fields per slide, were analyzed. The degree of histopathological alterations was evaluated using a semi-quantitative scoring system. There were four categories for histopathological scoring: none (−), mild (+), moderate (+ +), and severe (+ + +).

### DNA fragmentation assay

Following the manufacturer’s instructions, DNA was extracted from the spleen and liver homogenates using the AccuPrep^®^genomic DNA extraction kit (CAT. No. K-3032) and electrophoresed on an agarose gel that had been stained with ethidium bromide^35^. The ladder of the utilized DNA marker was 100–3000 bp, and it was inserted into the first well. Using Image J software (RRID: SCR_003070), the intensity of the lanes that could be seen on an agarose gel was measured^36^. The fold change was computed relative to the control group.

### Statistical analysis of data

The mean (*n* = 3) ± standard deviation was used to express all the data. Post-hoc multiple-comparison (Tukey) tests and one-way analysis of variance (ANOVA) were used to assess the data. A statistical difference of *p*≤ 0.05 was deemed significant. IBM SPSS Version 20.0, often known as the Statistical Package for the Social Sciences, was utilized to perform the statistical analyses^37^.

## Results

### Bioactive compounds in ***Sarcophyton*** crude extract

**Fig. 2:**
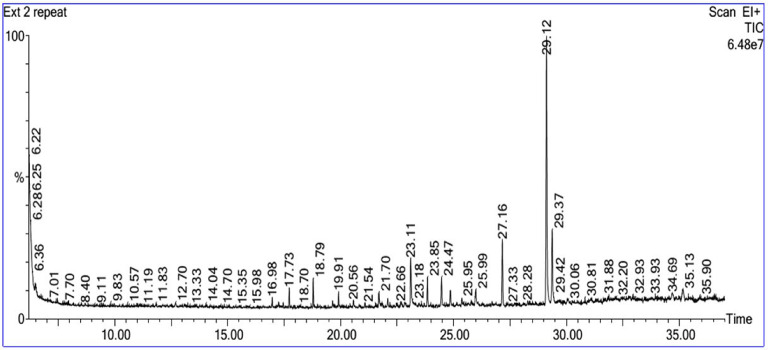
GC-MS chromatogram of *Sarcophyton* crude extract, displaying the compounds’ retention periods.

The GC-MS profile of the crude extract of *Sarcophyton* exhibited distinct chemical components at varying retention durations, as indicated by **Fig. 2** and **Table** **1**. In the *Sarcophyton* crude extract, vitamin A aldehyde was the most prevalent molecule, followed by isoaromadendrene epoxide, 3,7-cyclodecadien-1-one,3,7-dimethyl-10-(1-methylethylidene)-,(E, E)-, and palmitic acid, with peak area percentages of 15.604, 4.652, 3.339, and 2.968%, respectively **(Table** **1****)**.

**Table 1:** Chemical components identified by gas chromatography-mass spectrometry (GC-MS) in *Sarcophyton* crude extract.

Compound name	Compound nature	Molecular formula	MW (g/mol)	RT (min)	Height	Area (IU)	Area %
Fluoroacetic acid	Organofluorine compound	C_2_H_3_FO_2_	78.04	6.509	1,962,558	65881.9	0.343
1 H-Cycloprop[e]azulene, decahydro-1,1,7-trimethyl-4-methylene-, [1aR-(1aà,4aá,7à,7aá,7bà)]-	Sesquiterpene	C_15_H_24_O	220.35	16.978	2,527,396	73649.5	0.383
à-Cubebene	Sesquiterpene	C_15_H_24_	204.35	17.728	4,792,546	148339.6	0.771
Azulene, 1,2,3,3a,4,5,6,7-octahydro-1,4-dimethyl-7-(1-methylethenyl)-, [1R-(1à,3aá,4à,7á)]-	Sesquiterpene	C_15_H_24_	204.351	18.788	6,810,254	196896.9	1.024
Dodecane, 2,6,11-trimethyl-	Branched alkane	C_15_H_32_	212.41	19.914	3,878,918	111569.8	0.580
Myristic acid	Saturated fatty acid	C_14_H_28_O_2_	228.37	20.564	1,864,021	86049.9	0.447
Hexadecane	Alkane hydrocarbon	C_16_H_34_	226.44	21.079	1,402,690	54251.4	0.282
13-Tetradece-11-yn-1-ol	Fatty alcohol	C_14_H_24_O	208.34	21.700	3,520,176	181003.2	0.941
Stearyl alcohol	Fatty alcohol	C_18_H_38_O	270.5	22.095	2,150,164	94575.5	0.492
Sulfurous acid, nonyl 2-propyl ester	Ester	C_12_H_26_O_3_S	250.40	22.505	1,262,269	60111.8	0.313
Ursolic acid	Triterpene	C_30_H_48_O_3_	456.7	22.680	1,414,017	53688.6	0.279
Palmitic acid	Saturated fatty acid	C_16_H_32_O_2_	256.42	23.105	11,320,288	570755.4	2.968
ç-Elemene	Sesquiterpene	C_15_H_24_	204.35	23.845	6,801,325	241811.8	1.257
Thunbergol	Diterpene alcohol	C_20_H_34_O	290.5	24.471	6,725,182	280717.2	1.460
Bergamotol, Z-à-trans-	Fatty alcohol	C_15_H_24_O	220.350	24.861	3,701,910	160871.5	0.837
Andrographolide	Diterpene lactone	C_20_H_30_O_5_	350.4	25.366	2,076,321	71733.6	0.373
Octadecanoic acid	Saturated fatty acid	C_18_H_36_O_2_	284.4772	25.986	3,740,380	233049.2	1.212
3,7-Cyclodecadien-1-one, 3,7-dimethyl-10-(1-methylethylidene)-,(E, E)-	Sesquiterpene	C_15_H_22_O	218.334	27.162	15,443,440	642089.7	3.339
Vitamin A aldehyde	Vitamin aldehyde	C_20_H_22_D_6_O	290.47	29.118	61,543,108	3,000,798	15.604
Isoaromadendrene epoxide	Oxygenated Sesquiterpene	C_15_H_24_O	220.35	29.368	16,915,914	894617.7	4.652
Diazoprogesterone	Phytosterol	C_21_H_30_N_4_	338.489	30.033	1,639,573	50720.7	0.264
Etamiphyllin	Xanthine alkaloid	C_13_H_21_N_5_O_2_	279.34	30.078	1,421,383	74605.6	0.388
Benzeneethanamine, 2-fluoro-á,3-dihydroxy-Nmethyl-	Phenethylamine derivative	C_9_H_12_FNO_2_	185.2	31.113	1,624,501	56140.6	0.292
2,4(1 H,8 H)-Pteridinedione, 8-(2-hydroxyethyl)-	Pteridine derivative	C_8_H_8_N_4_O_3_	208.17	31.849	1,841,483	67211.0	0.350
Sinapic acid	Phenolic acid	C_11_H_12_O_5_	224.21	31.964	1,531,294	83576.6	0.435
Trimethyl(4-tert.-butylphenoxy)silane	Phenylalkoxysilane	C_13_H_22_OSi	222.399	32.204	1,618,527	71451.2	0.372
Cyclotrisiloxane, hexamethyl-	Organosilicon compound	C_6_H_18_O_3_Si_3_	222.46	32.244	1,357,247	51136.2	0.266
R-(-)-Cyclohexylethylamine	Secondary amine	C_8_H_17_N	127.23	32.484	1,600,821	56407.4	0.293
1,2-Benzisothiazol-3-amine tbdms	Benzothiazole secondary amine	C_13_H_20_N_2_SSi	264.46	32.549	1,683,236	102394.3	0.532
Benzenemethanol, 3-hydroxy-à-[(methylamino)methyl]-, (R)-	Amino alcohol	C_9_H_13_NO_2_	167.205	32.744	1,832,526	67541.2	0.351
2,4,6-Cycloheptatrien-1-one, 3,5-bis-trimethylsilyl-	Cyclic ketone	C_13_H_22_OSi_2_	250.48	32.784	1,773,136	85738.5	0.446
1,2,4-Benzenetricarboxylic acid, 1,2-dimethyl ester	Acid compound	C_11_H_10_O_6_	238.19	32.824	1,674,810	64030.4	0.333
1,2-Bis(trimethylsilyl)benzene	Hydrocarbon	C_12_H_22_Si_2_	222.47	32.904	1,457,265	57338.3	0.298
Cyclotetrasiloxane, octamethyl-	Cyclosiloxane	C_8_H_24_O_4_Si_4_	296.61	33.009	1,708,311	66008.7	0.343
Silane, 1,4-phenylenebis[trimethyl-	Organosilicon compound	C_12_H_22_Si_2_	222.47	33.674	1,210,816	54343.4	0.283
Benzene, 2-[(tert-butyldimethylsilyl)oxy]−1-isopropyl-4-methyl-	Organosilicon compound	C_16_H_28_OSi	264.48	33.984	1,526,260	62617.6	0.326
Ethyne, fluoro-	Organofluorine compound	C_2_HF	44.03	34.029	1,775,988	52,057	0.271
Imidazole, 2-amino-5-[(2-carboxy)vinyl]-	Imidazole carboxylic acid	C_6_H_7_N_3_O_2_	153.14	34.069	1,330,596	78324.2	0.407
Benzeneethanamine, 3-fluoro-á,5-dihydroxy-Nmethyl-	Organofluorine compound	C_9_H_12_FNO_2_	185.20	34.160	1,433,118	54498.8	0.283
Indole, 6-methyl-2-(2-pyridyl)-	Pyridine-indole compound	C_14_H_12_N_2_	208.26	34.230	1,522,934	53255.1	0.277
Medazepam	Benzodiazepine derivative	C_16_H_15_ClN_2_	270.75	34.465	1,269,869	84,088	0.437
Arachidonic acid	Unsaturated fatty acid	C_20_H_32_O_2_	304.5	34.690	2,348,467	242545.2	1.261
2’,6’-Dihydroxyacetophenone, bis(trimethylsilyl) ether	Ether	C_14_H_24_O_3_Si_2_	296.51	34.840	1,392,301	65704.1	0.342
Quinomethionate	Quinoxaline	C_8_H_6_N_2_OS_2_	210.276	34.945	940,980	52734.3	0.274
1,1,1,3,5,5,5-Heptamethyl-trisiloxane	Organosilicon compound	C_7_H_21_O_2_Si_3_	222.5	35.025	1,391,018	68126.7	0.354
cis-Z-à-Bisabolene epoxide	Sesquiterpene oxide	C_15_H_24_O	220.35	35.155	3,115,320	313805.2	1.632
2’,4’-Dihydroxyacetophenone, bis(trimethylsilyl) ether	Ether	C_14_H_24_O_3_Si_2_	296.509	35.430	1,238,285	63028.5	0.328
Ethanolamine	Primary amine and a primary alcohol	C_2_H_7_NO	61.08	35.900	1,502,453	57231.5	0.298

Molecular weight (MW) and Retention time (RT).

**Effect of** ***Sarcophyton*** **crude extract on body weight change**

The findings shown in **Table** **2** demonstrate that rats’ body weight change following oral administration of *Sarcophyton* extract (GII) was non-significantly different from that of the control group (GI). Rats injected with gentamicin saw a significant reduction in body weight (6.78%) as compared to control rats. Although the gentamicin side effect on the rat’s body weight was lessened by the combined administration of *Sarcophyton* extract and gentamicin (GIV), which caused an increase in body weight, there was still a significant difference from the control group (GI).

**Table 2:** The variations in body weight percentages in the different studied groups.

Groups	Percentage of body weight change (%)
**Control** **group** **(GI)**	16.06 ± 2.58^c^
***Sarcophyton*** **group** **(GII)**	14.29 ± 1.15^c^
**Gentamicin** **group** **(GIII)**	^_^6.78 ± 2.57^a^
**Combined administration** **group** **(GIV)**	8.89 ± 1.51^b^
**F-ratio**	60.71
***p*** **-value**	0.00

The value of each group represents the mean of three replicates ± standard deviation. Based on a one-way ANOVA, means in the same column with different superscript letters ^(a, b, and c)^ (*p*-value ≤ 0.05) are significantly different, and means in the same column with similar superscript letters (*p*-value > 0.05) are non-significantly different.

#### Hematological results

There was a non-significant difference in all hematological parameters between the oral administration of *Sarcophyton* extract (GII) and the control group (GI). Rats given gentamicin (GIII) had significantly lower RBCs, Hb, HCT%, MCHC, and Plts’ count than the control group (GI) but not significantly lower MCV or MCH. In addition, compared to the control group (GI), rats given gentamicin (GIII) showed a significant rise in WBC counts **(Table** **3****).**

**Table 3:** Complete blood counts (CBCs) in the different studied groups.

Test name Groups	RBCs’ count (M/µl)	Hb (g/dl)	HCT (%)	MCV (fl.)	MCH (pg)	MCHC (g/dl)	Plts’ count (10^3^/µl)	WBCs’ count (10^3^/µl)
**Control group** **(GI)**	6.20 ± 0.10^c^	16.56 ± 0.95^c^	50.66 ± 2.85^c^	81.60 ± 3.30^ab^	26.70 ± 1.10^ab^	32.66 ± 0.05^b^	602.66 ± 87.50^b^	6.06 ± 0.15^ab^
***Sarcophyton*** **group** **(GII)**	6.10 ± 0.10^c^	16.26 ± 0.45^b^^c^	51.06 ± 0.05^c^	82.33 ± 0.11^ab^	26.80 ± 0.17^ab^	32.63 ± 0.11^b^	691 ± 89^b^	5.80 ± 0.60^a^
**Gentamicin group** **(GIII)**	4.70 ± 0.10^a^	11.26 ± 1.05^a^	34.76 ± 3.15^a^	73.80 ± 5.10^a^	23.90 ± 1.70^a^	32.36 ± 0.05^a^	392.66 ± 12.50^a^	8.86 ± 0.75^c^
**Combined administration group** **(GIV)**	5.27 ± 0.23^b^	14.46 ± 0.15^b^	44.36 ± 0.45^b^	83 ± 3.20^b^	27.06 ± 1.05^b^	32.56 ± 0.05^b^	545 ± 24^ab^	7.56 ± 0.65^bc^
**F-ratio**	71.03	31.82	38.09	4.69	5.06	9.28	11.57	17.79
***p*** **-value**	0.00	0.00	0.00	0.03	0.03	0.006	0.003	0.001

Red blood cells (RBCs), Hemoglobin (Hb), Hematocrit (HCT), Mean corpuscular volume (MCV), Mean corpuscular hemoglobin (MCH), Mean corpuscular hemoglobin concentration (MCHC), Platelets (Plts), and White blood cells (WBCs). The value of each group represents the mean of three replicates ± standard deviation. Based on a one-way ANOVA, means in the same column with different superscript letters ^(a, b, and c)^ (*p*-value ≤ 0.05) are significantly different, and means in the same column with similar superscript letters (*p*-value > 0.05) are non-significantly different.

Combined administration of *Sarcophyton* extract with gentamicin (GIV) displayed significant decreases in RBCs, Hb, and HCT% and insignificant increases in MCV, MCH, and WBCs’ count, along with insignificant decreases in MCHC and Plts’ count compared to the control group (GI). However, there were significant increases in RBCs, Hb, HCT%, MCV, MCH, and MCHC, as well as an insignificant increase in Plts’ count in the combined administration group (GIV) compared with the gentamicin group (GIII). At the same time, the combined administration of *Sarcophyton* extract with gentamicin (GIV) showed an insignificant decrease in WBCs’ count compared with the gentamicin group (GIII) **(Table** **3****).**

#### Biochemical results

##### Liver enzyme activity

Oral administration of *Sarcophyton* extract (GII) produced non-significant variations in the activities of ALT and AST as compared to the control group (GI). Rats treated with gentamicin (GIII) showed significant increases in the activities of ALT and AST compared to the control group (GI) **(Table** **4****).**

**Table 4:** Activities of liver aminotransferase enzymes in the blood serum of different studied groups.

Groups	ALT (U/L)	AST (U/L)
**Control group** **(GI)**	23.90 ± 0.30^a^	36.86 ± 1.25^a^
***Sarcophyton*** **group** **(GII)**	24.06 ± 1.75^a^	33.06 ± 0.55^a^
**Gentamicin group** **(GIII)**	35.20 ± 1^c^	49.46 ± 2.75^b^
**Combined administration group** **(GIV)**	29.60 ± 2.30^b^	37.16 ± 2.65^a^
**F-ratio**	36.65	37.09
***p*** **-value**	0.00	0.00

Alanine amino transaminase (ALT) and Aspartate amino transaminase (AST). The value of each group represents the mean of three replicates ± standard deviation. Based on a one-way ANOVA, means in the same column with different superscript letters ^(a, b, and c)^ (*p*-value ≤ 0.05) are significantly different, and means in the same column with similar superscript letters (*p*-value > 0.05) are non-significantly different.

Combined administration of *Sarcophyton* extract with gentamicin (GIV) showed an insignificant increase in AST activity and a significant increase in ALT activity compared to the control group (GI). However, there were significant decreases in the activities of ALT and AST in the combined administration group (GIV) as compared with the gentamicin group (GIII) **(Table** **4****)**.

##### Lipid peroxidation

Oral administration of *Sarcophyton* extract (GII) did not significantly affect MDA levels in both the spleen and liver compared to the control group (GI). However, the administration of gentamicin (GIII) significantly increased the spleen’s and liver’s MDA levels compared to the control group (GI). Combined administration of *Sarcophyton* extract with gentamicin (GIV) resulted in significant increases in the spleen’s and liver’s MDA levels compared to the control group (GI). However, this combined administration group (GIV) showed significant decreases in the spleen’s and liver’s MDA levels compared to the gentamicin group (GIII) **(Table** **5****).**

**Table 5:** Levels of malondialdehyde (MDA) and total antioxidant capacity (TAC) in the spleen and liver tissues of the different studied groups.

Groups	Spleen		Liver	
	MDA (nmol/g. tissue)	TAC (mM/g. tissue)	MDA (nmol/g. tissue)	TAC (mM/g. tissue)
**Control group** **(GI)**	5.90 ± 0.30^a^	1.83 ± 0.05^c^	8.66 ± 1.05^a^	2.16 ± 0.05^c^
***Sarcophyton*** **group** **(GII)**	5.85 ± 0.65^a^	1.83 ± 0.15^c^	8.36 ± 0.55^a^	2.02 ± 0.05^c^
**Gentamicin group** **(GIII)**	14.46 ± 1.15^c^	1.07 ± 0.02^a^	21.60 ± 1.80^c^	1.02 ± 0.04^a^
**Combined administration group** **(GIV)**	10.96 ± 0.05^b^	1.36 ± 0.05^b^	13.16 ± 0.25^b^	1.63 ± 0.13^b^
**F-ratio**	115.12	56.84	97.005	129.39
***p*** **-value**	0.00	0.00	0.00	0.00

Malondialdehyde (MDA) and total antioxidant capacity (TAC). The value of each group represents the mean of three replicates ± standard deviation. Based on a one-way ANOVA, means in the same column with different superscript letters ^(a, b, and c)^ (*p*-value ≤ 0.05) are significantly different, and means in the same column with similar superscript letters (*p*-value > 0.05) are non-significantly different.

#### TAC

Oral administration of *Sarcophyton* extract (GII) did not significantly affect TAC levels in both the spleen and liver compared to the control group (GI). In contrast, the administration of gentamicin (GIII) significantly decreased the spleen’s and liver’s TAC levels compared to the control group (GI). Combined administration of *Sarcophyton* extract with gentamicin (GIV) showed significant decreases in the spleen’s and liver’s TAC levels compared to the control group (GI). However, this combined administration group (GIV) showed significant increases in the spleen’s and liver’s TAC levels compared to the gentamicin group (GIII) **(Table** **5****).**

##### Histological results of spleen tissue

**Fig. 3:**
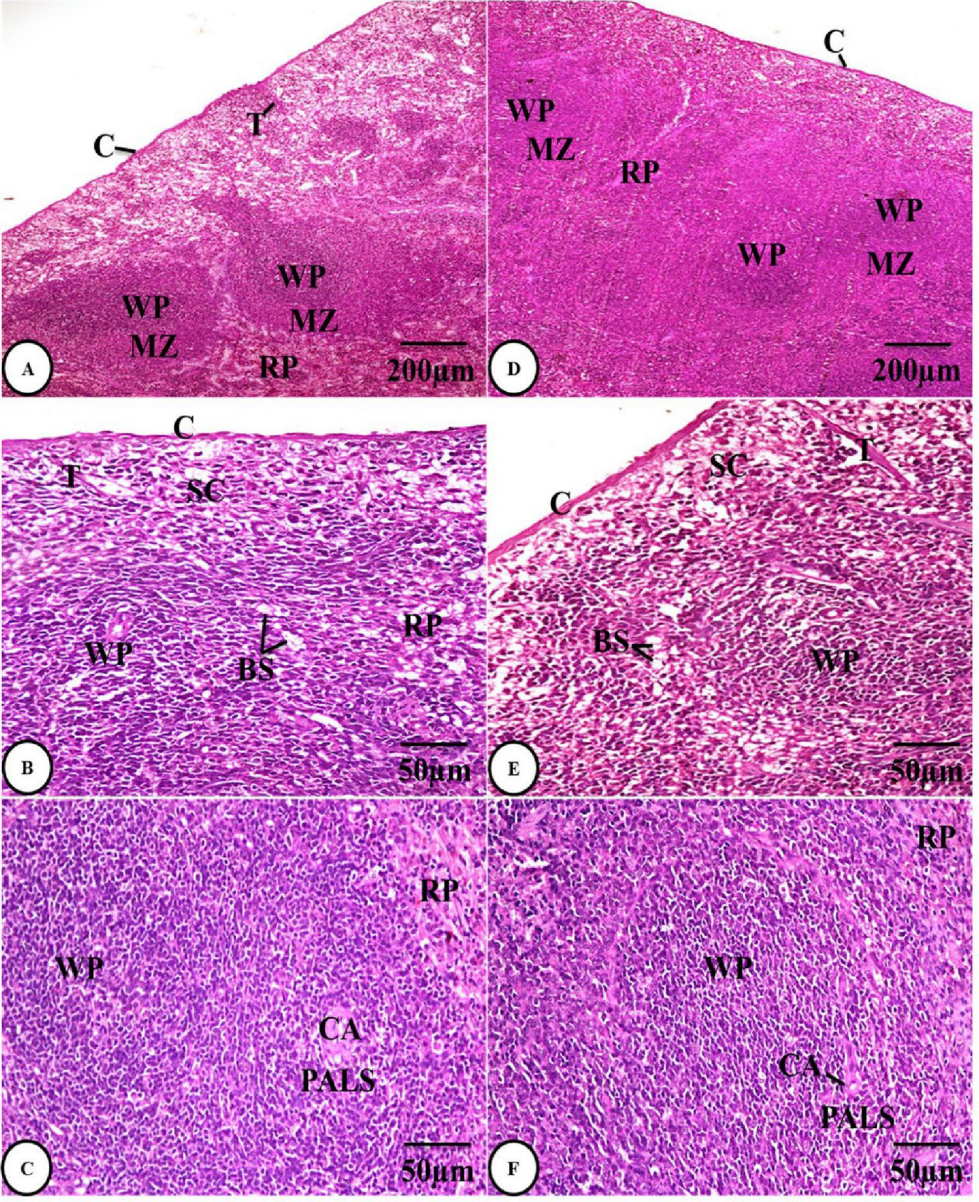
Photomicrographs of H&E-stained spleen sections of both the control group (GI) **(A-C)** and the *Sarcophyton* group (GII) **(D-F)** showing normal architecture with splenic capsules (C), trabeculae (T), red pulps (RP), white pulps (WP), marginal zones (MZ), blood sinusoids (BS), splenic cords (SC), and periarteriolar lymphoid sheaths (PALS) encircling central arterioles (CA).

Sections of the control group’s spleen were examined, and the results revealed normal architecture, which is described as the splenic pulp surrounded by a thin capsule of connective tissue that permits trabeculae to protrude through it and enter the splenic pulp’s parenchyma. This splenic pulp is divided into two portions, the red pulp and the white pulp, which are kept apart by a marginal zone. Blood sinusoids and splenic cords make up the red pulp, whereas lymphoid nodules and the periarteriolar lymphoid sheaths encircling central arterioles make up the white pulp **(Fig. 3A-C)**. Histologically, the *Sarcophyton* group (GII) had a considerable improvement **(Fig. 3D-F)**.

**Fig. 4:**
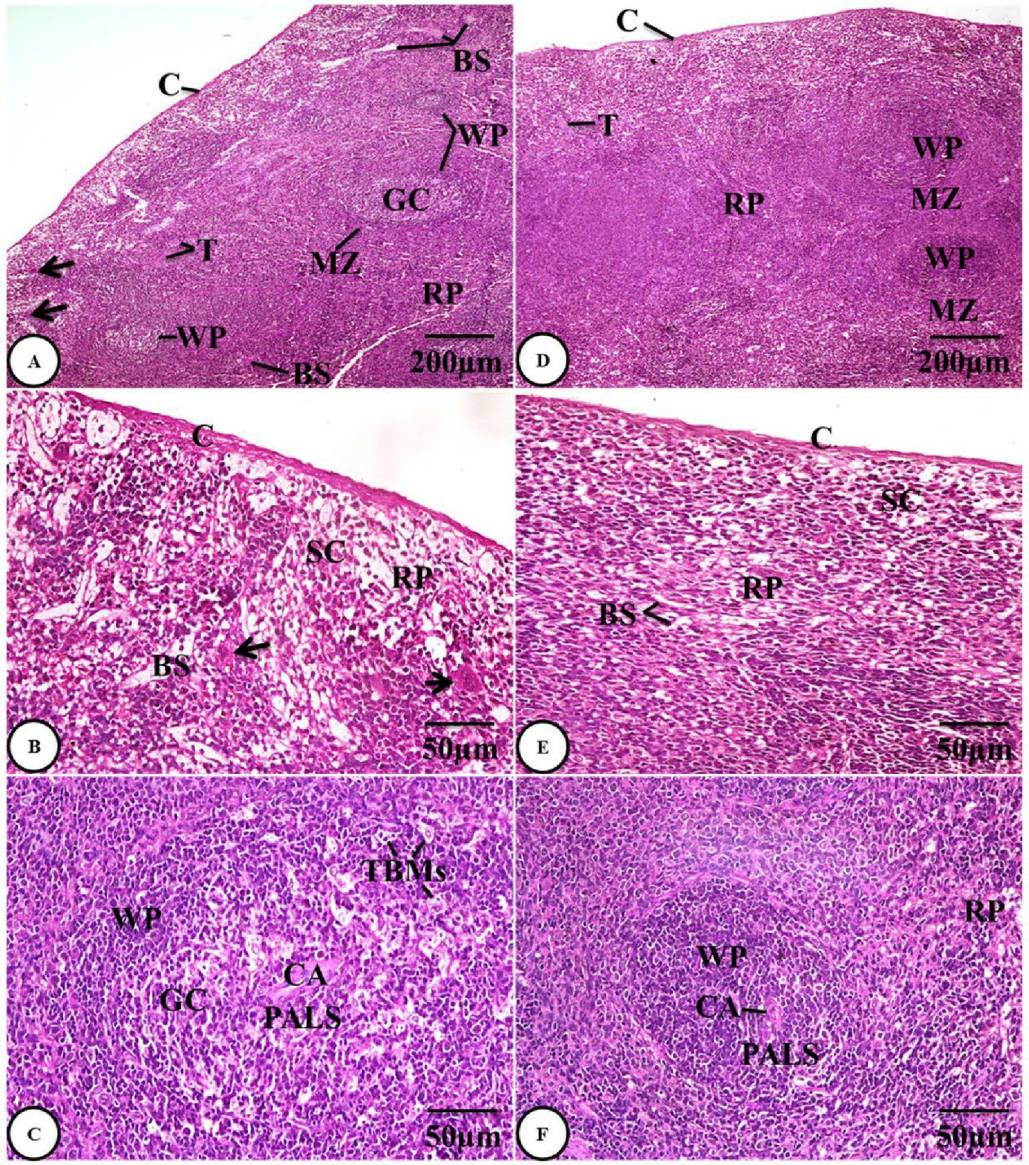
**A-C)** Photomicrographs of H&E-stained spleen sections of the gentamicin group (GIII) showing marked histological alterations as represented by an increase in the thickness of the splenic capsule (C), thicker trabeculae (T), red pulp (RP) containing dilated and congested blood sinusoids (BS), splenic cords (SC), and giant cells (arrows), disturbed lymphatic architecture of white pulp (WP) with the appearance of a germinal center (GC), containing multiple tingible body macrophages (TBMs) and periarteriolar lymphoid sheaths (PALS) encircling the central arteriole (CA) with a thicker wall, and the ill-defined marginal zone (MZ). **D-F)** Photomicrographs of H&E-stained spleen sections from the combined administration group (GIV) showing restoration of the normal architecture of the spleen tissues, normal thickness of the splenic capsule (C) and trabeculae (T), normal appearance of red pulps (RP) with blood sinusoids (BS) and splenic cords (SC), and regular white pulps (WP) with defined marginal zones (MZ) and periarteriolar lymphoid sheaths (PALS) encircling the normal central arteriole (CA).

Examination of spleen sections from the gentamicin group (GIII) showed marked histological alterations, as represented by an increase in the thickness of the splenic capsule and histological alterations in red pulps, which are evidenced by the dilated and congested splenic blood sinusoids and splenic cords, together with areas containing giant cells. A marked disturbance of the lymphatic architecture of the white pulp, with the appearance of a germinal center containing multiple tingible body macrophages with fragmented pyknotic nuclei, giving a starry sky appearance, was present. Additionally, a thicker wall of the central arteriole with fibrosis and smooth muscle cell proliferation was visible in the white pulp, and the marginal zones seemed vague and ill-defined **(Fig. 4A-C)**.

Examination of spleen sections from the combined administration group (GIV) revealed a notable improvement in the form of the splenic architecture, characterized by normal capsule thickness and trabeculae, a normal appearance of red pulp with blood sinusoids and splenic cords, and a regular white pulp structure with a clearly defined marginal zone and periarteriolar lymphoid sheaths encircling the normal central arteriole **(Fig. 4D-F)**.

##### Morphometric results of the spleen tissue

Oral administration of *Sarcophyton* extract (GII) produced non-significant variations in the capsule thickness and the lymphatic follicle’s diameter and surface area in comparison with the control group (GI). However, the administration of gentamicin (GIII) significantly increased these measurements compared to the control group (GI). The combined administration of *Sarcophyton* extract with gentamicin (GIV) was able to return capsule thickness and the lymphatic follicle’s diameter and surface area to their normal values **(Table ****6****).**

**Table 6:** Morphometric results of the spleen tissue in the different studied groups.

Groups	Capsule thickness (µm)	Lymphatic follicle diameter (µm)	Lymphatic follicle surface area (µm^2^)
**Control group** **(GI)**	36.07 ± 2.00^a^	930.63 ± 200.91^a^	1031077.22 ± 133672.11^a^
***Sarcophyton*** **group** **(GII)**	34.13 ± 4.35^a^	791.36 ± 101.69^a^	761410.73 ± 111632.44^a^
**Gentamicin group** **(GIII)**	92.34 ± 14.48^b^	1416.45 ± 212.50^b^	1900028.32 ± 205735.80^b^
**Combined administration group** **(GIV)**	31.49 ± 5.27^a^	825.07 ± 123.30^a^	747810.97 ± 212371.04^a^
**F-ratio**	39.45	9. 07	29.99
***p*** **-value**	0.00	0.006	0.00

The value of each group represents the mean of three replicates ± standard deviation. Based on a one-way ANOVA, means in the same column with different superscript letters ^(a and b)^ (*p*-value ≤ 0.05) are significantly different, and means in the same column with similar superscript letters (*p*-value > 0.05) are non-significantly different.

##### Histological results of liver tissue

**Fig. 5:**
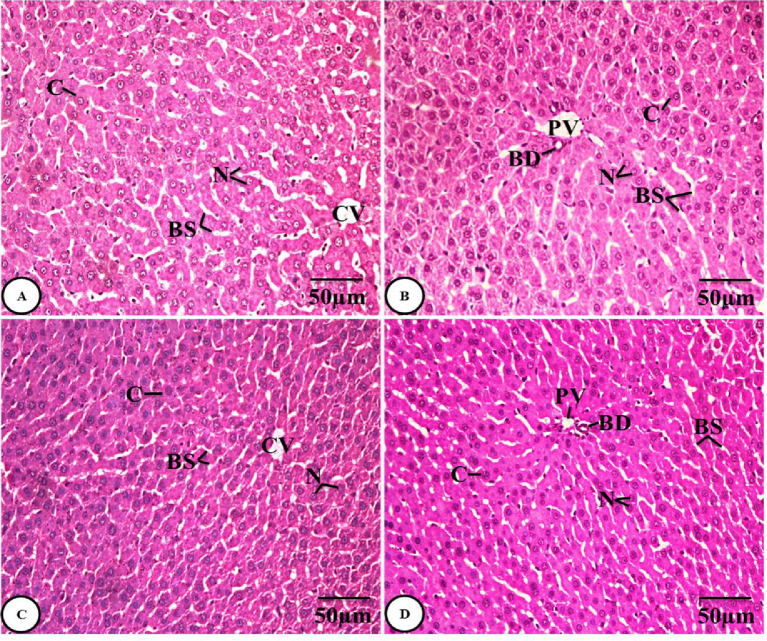
Photomicrographs of H&E-stained liver sections of both the control group **(A&B)** and the Sarcophyton group **(C&D)** showing normal liver tissue architecture with central veins **(CV)**, normal hepatocytes with round nuclei **(N)** and cytoplasm **(C)**, blood sinusoids **(BS)**, and portal areas with normal portal vein **(PV)** and bile duct **(BD)**.

**Fig. 6:**
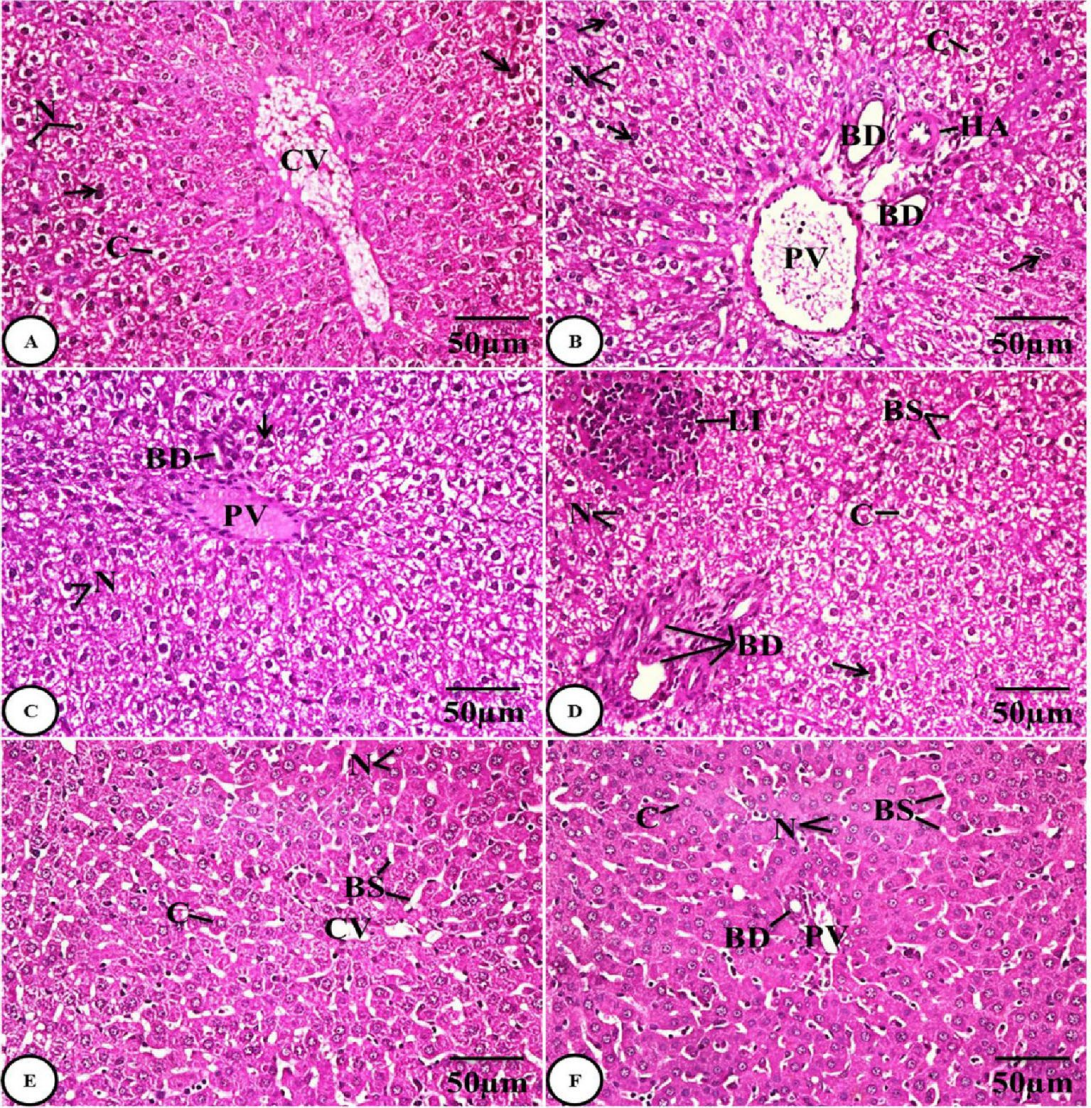
** A-D)** Photomicrographs of H&E-stained liver sections of the gentamicin group (GIII) showing disorganization of hepatic cords around the dilated central vein (CV), hepatocytic hyperplasia with binucleated cells (arrows), necrotic hepatocytes with vacuolated cytoplasm (C) and pyknotic nuclei (N), abnormal architecture of the portal area with a dilated and congested portal vein (PV), bile duct (BD) hyperplasia, thickened wall of the hepatic arteriole (HA), and inflammatory leukocytic infiltration (LI). **E&F)** Photomicrographs of H&E-stained liver sections from the combined administration group (GIV) showing restoration of the normal architecture of the liver tissues, characterized by normal hepatocytes with round nuclei (N) and cytoplasm (C), normal hepatocyte organization around the normal central vein (CV) and blood sinusoids (BS), and an improved portal area with a portal vein (PV) and bile duct (BD).

The examination of liver sections from the control group (GI) revealed a normal architecture of the liver tissue characterized by normally arranged hepatocytes radiating out from the central vein around the blood sinusoids and having round nuclei and cytoplasm **(Fig.** 5**A)**, along with a normal architecture of the portal area with a portal vein and bile duct lined with cuboidal cells **(Fig.** 5**B)**. The liver of the *Sarcophyton* group (GII) showed a considerable histological improvement **(Fig.** 5**C**, **D)**. The gentamicin group (GIII) liver sections underwent examination, and the results indicated hepatocellular injury in certain areas. This was indicated by the loss of the normal architecture of the liver tissues, which was characterized by dilation in the central vein, hepatic cord disarray, hepatocytic hyperplasia, which was indicated by an increase in the number of binucleated cells, and necrotic hepatocytes with hypertrophy, vacuolated cytoplasm, and pyknotic nuclei. Moreover, the abnormal architecture of the portal area with a dilated and congested portal vein, bile duct hyperplasia, thickened wall of the hepatic arteriole, and inflammatory leukocytic infiltration was observed **(Fig.** 6**A-D)**.

Examination of liver sections from the combined administration of *Sarcophyton* extract with gentamicin group (GIV) showed restoration of the normal architecture of the liver tissues, characterized by normal hepatocytes with round nuclei and cytoplasm, radiating out from the central vein around the blood sinusoids **(Fig. 6E)**, and an improved portal area with a portal vein and bile duct **(Fig. 6F)**.

#### Histopathological scoring analysis of liver sections

**Table** **7** provides a summary of the graded liver histopathology score results. No histopathological changes were observed in the control, *Sarcophyton*, and combined administration groups, while the gentamicin group showed moderate histopathological changes like sinusoidal dilation, vacuolated cytoplasm, cellular necrosis, and leukocytic infiltration, and severe histopathological changes, such as disorganized hepatic cords, central vein dilation, and pyknotic nuclei.

**Table 7:** Histopathological scoring analysis of liver sections of the different studied groups.

Histological scoring	Control group (GI)	*Sarcophyton* group (GII)	Gentamicin group (GIII)	Combined administration group (GIV)
**Disorganized hepatic cords**	**−**	**−**	**+ + +**	**−**
**Central vein dilation**	**−**	**−**	**+ + +**	**−**
**Sinusoidal dilation**	**−**	**−**	**+ +**	**−**
**Pyknotic nuclei**	**−**	**−**	**+ + +**	**−**
**Vacuolated cytoplasm**	**−**	**−**	**+ +**	**−**
**Cellular necrosis**	**−**	**−**	**+ +**	**−**
**Leukocytic infiltration**	**−**	**−**	**+ +**	**−**

None (−), mild (+), moderate (+ +), and severe (+ + +) changes.

#### DNA fragmentation

**Fig. 7:**
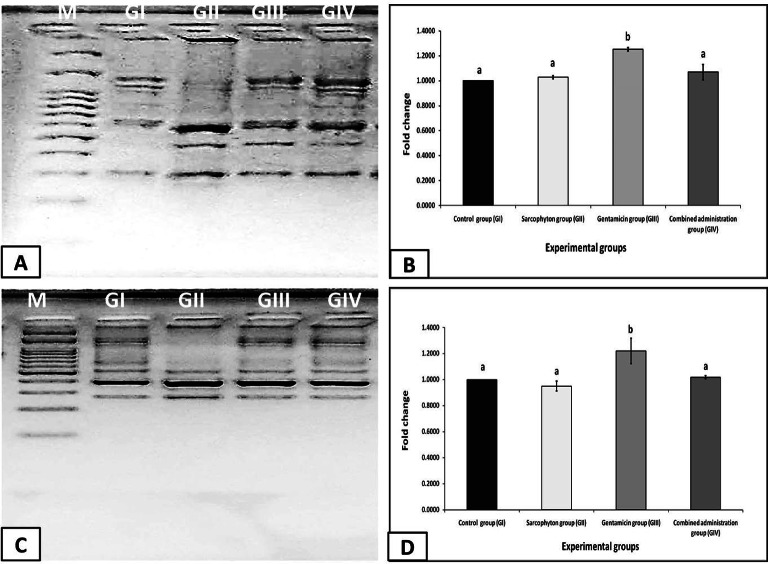
DNA fragmentation of the spleen **(A&B)** and liver **(C&D)** tissues in the different studied groups in a comparison with the DNA marker (M) ranging from 100 to 3000 bp. The value of each bar represents the mean of three replicates ± standard deviation. Based on a one-way ANOVA, different letters ^(a and b)^ (*p*-value ≤ 0.05) are significantly different, and similar letters (*p*-value > 0.05) are non-significantly different.

After the oral administration of *Sarcophyton* extract (GII), the level of DNA fragmentation in the spleen and liver tissues was non-significant compared to the control group (GI). However, the administration of gentamicin (GIII) significantly increased the level of DNA fragmentation in both tissues compared to the control group (GI). At the same time, the combined administration of *Sarcophyton* extract with gentamicin (GIV) resulted in non-significant increases and significant decreases in the spleen’s and liver’s DNA fragmentation levels compared to the control group (GI) and the gentamicin group (GIII), respectively **(Fig. 7).**

## Discussion

Gentamicin is a commonly used aminoglycoside antibiotic drug with beneficial antimicrobial effects against life-threatening gram-negative bacteria^38^. Despite it’s wide application in clinical practice, it’s prescription has been limited due to serious side effects on the kidney, liver, and other body organs^39,40^.

The progression of oxidative stress is regarded as the main mechanism contributing to the toxicity induced by gentamicin therapy^40,41^. Therefore, various previous studies have focused on using antioxidant medications obtained from natural sources to provide protection against gentamicin’s deleterious effects and ameliorate it’s toxicity^3,42–44^. Although it is known that *Sarcophyton *has antioxidant compounds of different classes like fatty acids, sesquiterpenes, and diterpenes^45^, no studies have been performed on it yet to explore it’s effect on the spleen and liver against gentamicin toxicity. Therefore, this study demonstrates, for the first time, the potential ameliorative effect of *Sarcophyton* extract on gentamicin-induced splenotoxicity and hepatotoxicity.

The current findings suggest that gentamicin can have an impact on rats’ body weight by significantly lowering it in comparison to control rats. The combined administration of *Sarcophyton* extract and gentamicin (GIV) reduced the negative effect of gentamicin on body weight by assisting in the decrease of body weight loss. In the current investigation, the ameliorative effect of *Sarcophyton *extract was validated by a non-significant difference in the body weight change between GII rats and GI control rats. These outcomes are consistent with earlier research on the effects of gentamicin-induced hepatotoxicity and nephrotoxicity on body weight^46–48^. The notable reduction in body weight observed in the gentamicin group may be attributed to increased catabolism and anorexia, both of which reduce food intake and result in weight loss. Moreover, the loss of tubular cells involved in renal water absorption causes the body to lose weight and become dehydrated^49^. **Al-Khamasear et al.**^49^, who studied the hepatoprotective activity of berberine at doses of 50 and 100 mg/kg body weight against gentamicin 80 mg/kg-induced hepatic toxicity in rats for seven days, observed that gentamicin reduced appetite, decreased activity, and caused progressive physical fatigue, whereas the treatment with berberine at both doses produced significant improvement in body weight compared to gentamicin-induced rats, similar to our findings in this study following the oral administration of *Sarcophyton* at a dose of 200 mg/kg/day for seven days.

**Fig. 8:**
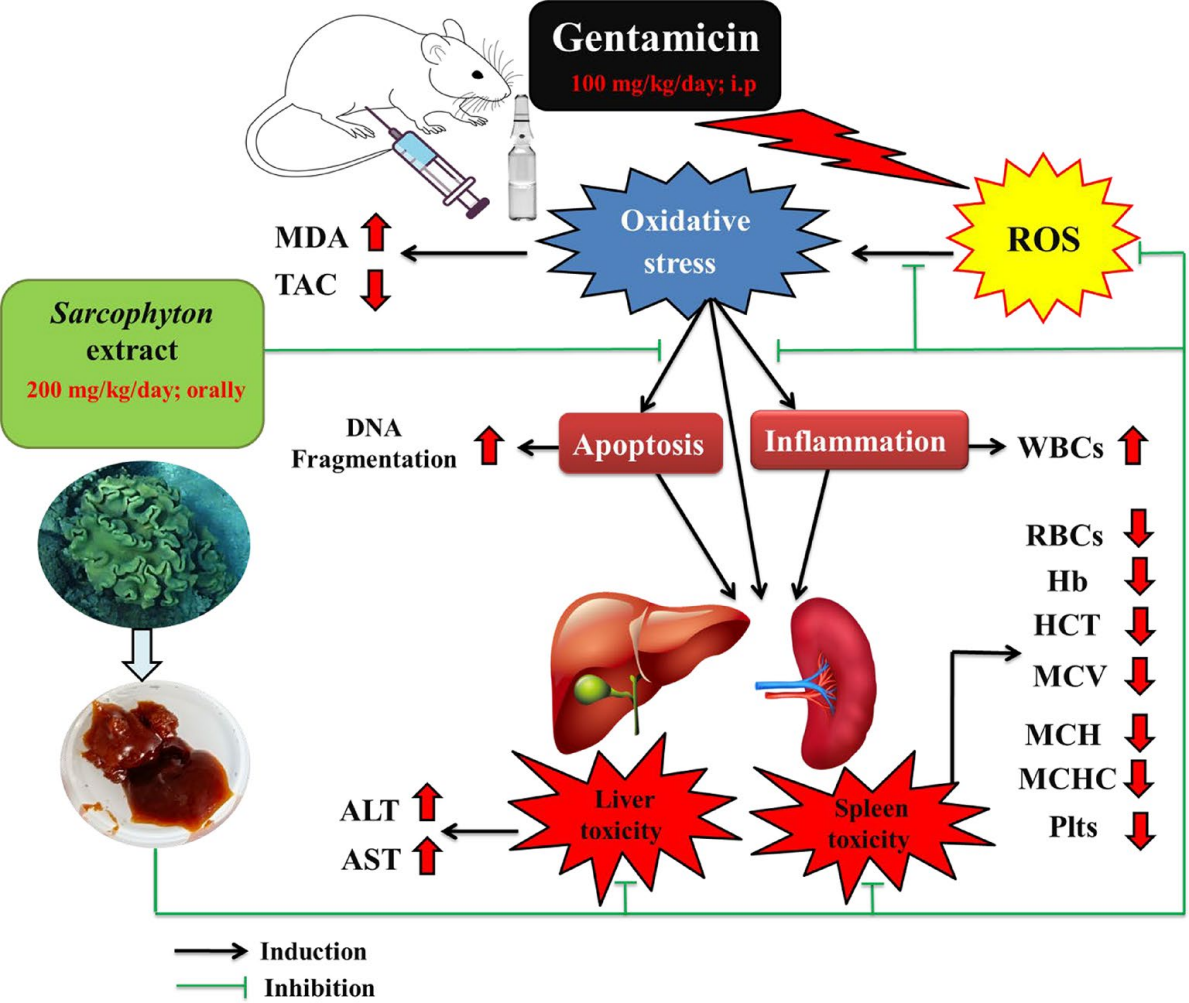
Diagram showing mechanisms of action of gentamicin and the ameliorative effect of *Sarcophyton* extract.

In the current work, gentamicin toxicity was evaluated through alterations in the biochemical indicators of oxidative stress, mainly MDA and TAC. MDA is widely used as an indicator marker to evaluate lipid peroxide formation because it is known as the final product of lipid peroxidation and is associated with increased ROS levels^50^. The injection of rats with gentamicin (GIII) significantly augmented MDA levels in the tissues of the spleen and liver, accompanied by a significant drop in the TAC in both tissues compared to the control rats (GI) **(Fig. 8)**. The increase of MDA level and decreasing of antioxidant capacity in the tissues can be explained by the ability of gentamicin to induce changes in redox status, causing oxidative stress and lipid peroxidation accompanied by the overgeneration of ROS, such as hydroxyl radicals (^•^OH), hydrogen peroxide (H_2_O_2_), and superoxide anions (O_2_^•─^), and the depletion of antioxidant enzymes in combating oxidative stress^39,51,52^. This outcome is in harmony with those of **Yarijani et al.**^3^, **Bekheet et al.**^53^, and **Wijayanti et al.**^54^, who showed that gentamicin administration significantly increased MDA levels and significantly declined antioxidant status levels in tissues as compared with the control group.

In contrast, co-administration of *Sarcophyton* extract with gentamicin (GIV) displayed inverse results in MDA and TAC levels. It significantly declined the MDA levels and significantly upregulated the TAC in the spleen and liver tissues compared to the gentamicin group (GIII), indicating that *Sarcophyton* extract reduces oxidative stress *via* the free radical scavenging abilities of the antioxidant compounds detected in it by GC-MS analysis in the present study **(Fig. 8)**. These compounds include cyclotetrasiloxane, octamethyl-, cyclotrisiloxane, hexamethyl-^27^, octadecanoic acid^55^, vitamin A aldehyde^56,57^, ursolic acid^58,59^, sinapic acid^60–62^, isoaromadendrene epoxide^63^, palmitic acid^64,65^, hexadecane^66^, myristic acid^66,67^, 2,4,6-cycloheptatrien-1-one,3,5-bis-trimethylsilyl-^68^, thunbergol^69^, and 3,7-cyclodecadien-1-one,3,7-dimethyl-10-(1-methylethylidene)-,(E, E)-^70^. Moreover, the antioxidant properties of the *Sarcophyton* extract were confirmed in the current work by non-significant variations in the MDA and TAC levels in both the spleen and liver tissues after the oral administration of the *Sarcophyton* extract (GII) compared with those of the control group (GI). The *Sarcophyton* findings in the present study are in line with those of the prior studies, which indicated that antioxidant-based treatments like *Moringa oleifera *seed oil at an oral dose of 5 ml/kg body weight for 16 days and boiled water spinach at a dose of 20 g/rat/day for seven days alleviated lipid peroxidation and enhanced antioxidants in gentamicin-induced rats^71,72^. Additionally, **Abdel-Wahhab et al.**^18^ found that the administration of sarcophine terpenoid (20 mg/kg body weight for 2 weeks), which was isolated from the soft coral *Sarcophyton*, had antioxidant effects on liver tissues through the significant reduction in MDA and enhancement of TAC in the serum of CCl_4_-intoxicated rats (1 mg/kg body weight for 2 weeks).

Hematological parameters are important indicators of physiological and pathological status and provide important information about abnormalities and toxic effects^73^. In the current research, the administration of gentamicin showed significant decreases in RBCs, Hb, HCT%, MCHC, and Plts’ count **(Fig. 8)**. The lowering of RBCs and HCT, as observed in our results, indicates the insufficient flow of blood to the tissues, which impedes the physiological transport of oxygen and carbon dioxide, causing an anemic state^74,75^. The drop in RBCs’ count might be related to a decrease in erythropoiesis in the bone marrow and a higher rate of destruction of peripheral RBCs in the spleen^76^. Cellular damage to RBCs may be due to lipid peroxidation, membrane protein cross-linking, fragmentation induced by free radicals^77^, and decreasing the release of erythropoietin, the humoral regulator of RBCs’ production, from the kidneys^75^.

Hemoglobin is the substance that gives RBCs color and facilitates oxygen transport throughout the body, and it’s low level can result in insufficient oxygen supply^78^. Also, it is known as a metalloprotein found in RBCs and can be chelated by gentamicin, forming an iron‒gentamicin complex, a powerful catalyst for generating free radicals^79^. A decrease in the Plts’ count (thrombocytopenia) after administering gentamicin (GIII) can impede blood clotting and increase the risk of severe bleeding^80^. Similar outcomes were documented by **Simeon et al.**^81^, who recorded a decrease in RBCs, Hb, HCT%, and Plts’ count in rats medicated with gentamicin (100 mg/kg body weight daily for 8 days).

In this study, oral *Sarcophyton* extract administration (GII) produced a non-significant variation in all hematological parameters compared with those of control rats (GI), and the combined administration of *Sarcophyton* extract with gentamicin (GIV) increased RBCs, Hb, HCT%, MCV, MCH, MCHC, and Plts’ count compared with those of the gentamicin group (GIII). Moreover, the obtained results agree with the previous study of **Hamdy et al.**^8^, who studied the effect of curcumin (200 mg/kg body weight, orally for 21 days) in protecting against gentamicin (100 mg/kg body weight, intraperitoneally, daily for seven days) and reported that the gentamicin-intoxicated group showed a marked decline in RBCs’ count and Hb concentration, as well as HCT%, with insignificant differences in MCV, MCH, and MCHC. At the same time, the administration of curcumin with gentamicin caused improvement in all hematological parameters, similar to the results of the combined administration group (GIV) reported in the present study. This improvement in hematological parameters after *Sarcophyton* extract administration may be related to the antioxidant chemical compounds detected in the *Sarcophyton* extract by GC-MS analysis in the present study, which may be a clue to it’s ability to protect blood cell membranes from oxidation, thus preventing cellular damage **(Fig. 8)**.

Leukocytes have many functions, particularly in the recognition, metabolism, and elimination of xenobiotics, and WBCs’ count is considered a clinical inflammatory marker^82^. As regards WBCs’ count in the present study, the rats treated with gentamicin (GIII) exhibited a significant elevation in WBCs’ count compared to the control rats (GI) **(Fig. 8)**. The toxic metabolites that induce liver damage may trigger an innate immune response, leading to the activation of leukocytes and dysregulation of immune cell activity in trying to protect the body from being vulnerable to infections, as reported in previous studies after oral toxicant administration^76,83,84^. Also, renal ischemia-reperfusion induced by gentamicin may lead to an increase in leukocyte infiltration^85,86^. Similar results were reported by **Talaat et al.**^84^, who studied the effect of frankincense oil against paracetamol toxicity, and they reported that administration of paracetamol led to an increase in total WBCs’ count.

At the same time, co-administration of *Sarcophyton* extract with gentamicin to rats (GIV) induced a reduction in the WBCs’ count relative to that in the gentamicin group (GIII), although it did not normalize it. The ameliorative effect of *Sarcophyton* extract on WBCs’ count was evidenced by the non-significant variation in WBCs’ count after oral administration of *Sarcophyton* extract (GII) compared with that in control rats (GI). This is explained by the anti-inflammatory qualities of the chemical compounds detected in the *Sarcophyton *extract in the present study by GC-MS analysis, like vitamin A aldehyde^87^, sinapic acid^88–90^, andrographolide^91,92^, palmitic acid^93^, ursolic acid^94–96^, and myristic acid^97^ **(Fig. 8)**. **Talaat et al.**^84^ reported similar findings, where natural products of frankincense oil reverse paracetamol toxicity by decreasing total WBCs’ count.

A splenic histopathology examination is highly recommended for immune system evaluation^98^. In the present histological study, the spleen tissue of the rats administered gentamicin (GIII) underwent noteworthy histological changes, as evidenced by a significant rise in the thickness of the splenic capsule, a significant rise in the diameter and surface area of the lymphatic follicles, the presence of dilated and congested blood sinusoids and giant cells in the red pulp, and a disturbed lymphatic architecture of white pulp with the appearance of a reactive germinal center containing multiple tingible body macrophages and hypertrophic central arterioles, with fibrosis encircled by periarteriolar lymphoid sheaths. These splenic histological abnormalities may have been caused by gentamicin-induced oxidative stress. Also, the appearance of giant cells is thought to be caused by inflammation induced by gentamicin because when monocytes move to the site of inflammation, they mix with macrophages to form multinucleated giant cells^99^. These results are in line with **Udo**’s spleen histology findings^100^, which demonstrated how a toxicant like paracetamol can promote lymphoid follicular hyperplasia, with an indication that spleen histopathology can be associated with a number of other organ disorders, including kidney illness, and have a deleterious effect on the immune system by causing the mobilization of lymphoid cells into body tissues when the toxicant is present.

The current histological examination of the spleen tissue after the combined administration of *Sarcophyton* extract with gentamicin (GIV) showed restoration of the typical architecture of the spleen tissues with normal capsule thickness and the lymphatic follicle’s diameter and surface area, which may be due to the antioxidant and anti-inflammatory compounds in the *Sarcophyton *extract. Analogous results have been documented in other research, wherein natural extracts have demonstrated the capacity to counteract gentamicin toxicity, thereby augmenting the immune system’s defensive capacity^78^.

The blood serum activities of ALT and AST enzymes were estimated to assess liver function^101,102^. The present research revealed that rats receiving gentamicin (GIII) had a severe liver injury, as presented by a marked rise in ALT and AST activities compared with those in control rats **(Fig. 8)**. Similar findings were obtained by **Laaroussi et al.**^1^, **Khaksari et al.**^39^, **Yarijani et al.**^103^, and **Elgazzar et al.**^104^. The increase in the analyzed enzymes is indicative of hepatotoxicity induced by gentamicin through oxidative stress, causing hepatic cell necrosis and structural alterations in the membrane permeability of liver cells that lead to the enzymes being secreted into the circulation from the cytosol^3,39,104^. This theory is backed by the liver histopathological alterations seen in the present study of the rats given gentamicin (GIII), including central vein dilation, hepatocytic hyperplasia, necrotic hepatocytes with hypertrophy, vacuolated cytoplasm, and pyknotic nuclei, and an abnormal architecture of the portal area with a dilated and congested portal vein, bile duct hyperplasia, a thickened wall of the hepatic arteriole, and inflammatory leukocytic infiltration. The liver histology results obtained are in harmony with the findings published by **Inam et al.**^105^ and **Metin**^106^.

However, liver enzyme activities significantly declined in the combined administration group (GIV) compared to the gentamicin group (GIII). This result aligns with the findings of **Laaroussi et al.**^1^, who showed a preventive effect of propolis (100 mg/kg body weight) or honey (2 g/kg body weight) after their coadministration with gentamicin (120 mg/kg body weight/day, intraperitoneally) for 10 days by decreasing the plasmatic AST and ALT activity levels in rats. Additionally, **Yarijani et al.**^103^ demonstrated that the concomitant use of date palm pollen extract at dosages of 200 or 400 mg/kg for nine consecutive days with gentamicin at a dosage of 100 mg/kg intraperitoneally from the third to the ninth day significantly reduced AST and ALT activity levels in rats as compared to the gentamicin group. The present findings of the histological examination of the liver tissue after the combined administration of *Sarcophyton* extract with gentamicin (GIV) confirmed these biochemical results and showed the restoration of the typical architecture of the liver tissues. Our histological observations were consistent with those of **Tammam et al.**^17^, who showed that the liver tissue of the group treated with *Sarcophyton glaucum* soft corals had almost normal structure. These findings suggest that *Sarcophyton* extract offers protection to liver cells through it’s antioxidant bioactive compounds and does not have a necrotic effect on liver tissue **(Fig. 8)**. Hence, it can reduce oxidative stress and decrease damage to hepatocyte membranes, leading to a reduction in enzyme leakage. Also, GC-MS analysis in the present study detected some hepatoprotective agents like andrographolide^107,108^, ursolic acid^109,110^, and sinapic acid^111,112^ in the *Sarcophyton* crude extract. This finding is consistent with **Zidan et al.**^11^ and **Abdel-Wahhab et al.**^18^, who revealed that soft coral *Sarcophyton* extract has hepatoprotective activity through decreasing ALT and AST activities in the serum of CCl_4_-intoxicated rats.

Compared with the control group, the spleen and liver tissues in the present study had significantly high levels of DNA fragmentation after the rats received gentamicin (GIII) **(Fig. 8)**. DNA fragmentation is a crucial indicator of DNA damage caused by physical or chemical stresses, particularly when cytoprotection mechanisms are insufficient^113,114^. Also, it can occur due to genotoxic insults, oxidative damage, or DNA replication errors, leading to genomic instability and potential genetic disorders or cancer^115^. Gentamicin can cause apoptosis by elevating oxidative stress, which explains why the intensity of DNA fragmentation in the spleen and liver tissues was elevated. As shown in prior work, gentamicin attacks mitochondria, leading to preventing respiration and causing oxidative stress in order to trigger the intrinsic route of apoptosis^116^. Subsequently, certain proteases known as executioner caspases (caspase-3 and -7) are activated, leading to the manifestation of typical apoptotic morphological indicators such as cellular shrinkage and DNA fragmentation^117^. The present results of DNA fragmentation confirmed the histopathological findings of splenic toxicity and hepatocellular damage induced by gentamicin. The current findings are consistent with earlier research showing that tissue damage caused by gentamicin was validated by DNA fragmentation analysis^100,118^.

However, co-administration of *Sarcophyton* extract with gentamicin to rats (GIV) in the present study produced a protective impact on the DNA of the spleen and liver tissues. In fact, the amount of DNA fragmentation following oral administration of *Sarcophyton* extract (GII) was not significantly different from that of control rats (GI), indicating that the extract had a mitigating impact on DNA damage. This can be attributed to the chemical components’ antioxidant properties found in the *Sarcophyton* extract, detected in this study by GC-MS analysis, which in turn lessen DNA oxidative damage **(Fig. 8)**. In agreement with the present obtained results, **Hegazy et al.**^118^, who studied the effects of aqueous extracts of rosemary or thyme on the gentamicin-induced hepatotoxicity in rats, indicated that DNA fragmentation in liver tissue was markedly reduced after 10 days of oral administration of both extracts at a dosage of 10 ml/kg/day.

## Conclusion

Our present study concluded that *Sarcophyton* extract can be a potential natural drug for ameliorating gentamicin-induced toxicity. It can reduce MDA generation and improve TAC in tissues *via* it’s contents of antioxidant and anti-inflammatory compounds, thus helping maintain body weight, hematological parameter levels, and liver enzyme activities within normal ranges, restoring the spleen and liver histological architecture, and decreasing DNA fragmentation level in the tissues. Thus, the present work, for the first time, lays the groundwork for developing *Sarcophyton*-based treatments for practical applications, particularly in mitigating side effects of aminoglycoside antibiotics like gentamicin. However, further studies are needed to provide new insights into the molecular mechanisms underlying the action of *Sarcophyton* extract in the treatment of gentamicin-induced toxicity, and future studies for the purification of *Sarcophyton*’s bioactive compounds are necessary for clinical application.

## Data Availability

The research data used to support the findings of this study are included within the article (tables, figures).
